# Multiperspective Light Field Reconstruction Method via Transfer Reinforcement Learning

**DOI:** 10.1155/2020/8989752

**Published:** 2020-02-14

**Authors:** Lei Cai, Peien Luo, Guangfu Zhou, Tao Xu, Zhenxue Chen

**Affiliations:** ^1^School of Artificial Intelligence, Henan Institute of Science and Technology, Xinxiang 453003, China; ^2^School of Information Engineering, Henan Institute of Science and Technology, Xinxiang 453003, China; ^3^School of Control Science and Engineering, Shandong University, Jinan 250061, China

## Abstract

Compared with traditional imaging, the light field contains more comprehensive image information and higher image quality. However, the available data for light field reconstruction are limited, and the repeated calculation of data seriously affects the accuracy and the real-time performance of multiperspective light field reconstruction. To solve the problems, this paper proposes a multiperspective light field reconstruction method based on transfer reinforcement learning. Firstly, the similarity measurement model is established. According to the similarity threshold of the source domain and the target domain, the reinforcement learning model or the feature transfer learning model is autonomously selected. Secondly, the reinforcement learning model is established. The model uses multiagent (i.e., multiperspective) Q-learning to learn the feature set that is most similar to the target domain and the source domain and feeds it back to the source domain. This model increases the capacity of the source-domain samples and improves the accuracy of light field reconstruction. Finally, the feature transfer learning model is established. The model uses PCA to obtain the maximum embedding space of source-domain and target-domain features and maps similar features to a new space for label data migration. This model solves the problems of multiperspective data redundancy and repeated calculations and improves the real-time performance of maneuvering target recognition. Extensive experiments on PASCAL VOC datasets demonstrate the effectiveness of the proposed algorithm against the existing algorithms.

## 1. Introduction

Light field [1, 2] is the parametric representation of a 4D light radiation field that contains the position and direction information in space. In other words, the light field contains all images of the same object taken at different positions and different angles.

The light field can be used as a feature library of the target images. The multiperspective light field is a complete light field represented by multiple perspectives. The multimodal fusion of the captured target information is performed through the cooperation mechanism among different perspectives, and finally, the dataset of the multiperspective light field is obtained. With the rapid development of light field reconstruction technologies, the target recognition employing the light field feature library has been widely studied in the fields of computer vision, pattern recognition, and image processing. In the military field, the target recognition is applied to counterterrorism, missile guidance, search and rescue, and target monitoring in the airspace. In the industrial field, the target recognition is employed to the robot navigation, industrial part detection, and assembly line. In the civil field, the target recognition is applied to biomedicine, intelligent transportation, and motion tracking. Modal perception in the area of the computer is a channel that uses computer technology to imitate human beings and make machines connect with the physical world. Environment segmentation is to use computer technology to segment the acquired environment according to different categories. Finally, the formal representation and description of the segmented image are called feature representation. With the popularization of artificial intelligence technology, many learning methods have been widely investigated, for example, reinforcement learning [3], transfer learning [4], and antagonistic learning [5]. The successful application of artificial intelligence technology in the field of image recognition is no more than deep learning [6]. In recent years, researchers have trained image samples through a convolution neural network [7] and other deep learning models, which improve the accuracy of target recognition.

**Figure 1:**
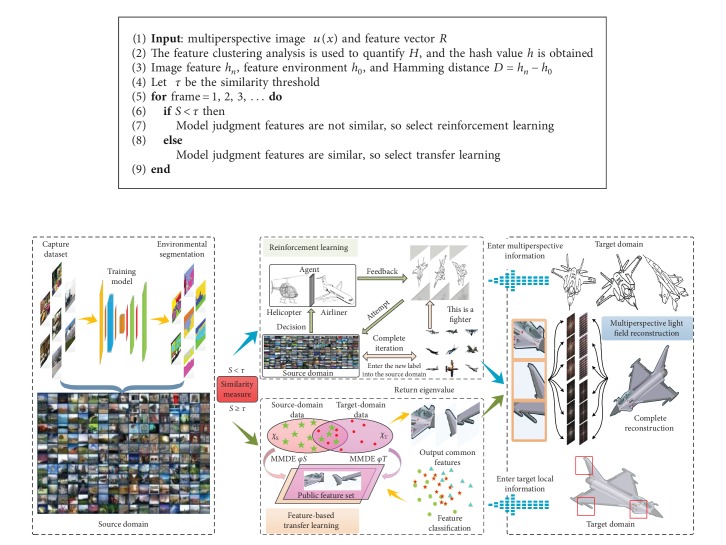
Overall flow chart of the multiperspective light field reconstruction method via transfer reinforcement learning.

However, the complex computing process seriously affects the real-time performance and accuracy of target recognition. To solve the problem, this paper proposes the multiperspective light field reconstruction method based on transfer reinforcement learning, as shown in Figure 1.

The transfer reinforcement learning algorithm is free from the constraints of large data and large sample training. The transfer learning and reinforcement learning are selected independently according to similarity thresholds based on the samples of tags with limited source domains.

The main contributions of this paper are as follows:

1. This paper introduces reinforcement learning and transfer learning algorithms into the area of light field reconstruction. According to the similarity measure model, the reinforcement learning and transfer learning are autonomously chosen.
2. The reconstruction method for the multiperspective light field via transfer reinforcement learning effectively reduces the calculation of similar data in the reconstruction process, shortens the time of light field reconstruction, and improves the real-time performance of target recognition.
3. This paper conducts extensive experiments to evaluate the performance of the proposed method in target recognition. The experimental result shows that this method is superior to existing methods.

The remainder of this paper is organized as follows: Section 2 provides an overview of the related literature.  Section 3 describes the multiperspective representation and source-domain establishment of the light field, the transfer reinforcement learning algorithm, and the reconstruction method for the multiperspective light field based on transfer reinforcement learning. The simulation experiments are discussed in Section 4. Finally, Section 5 concludes this paper.

## 2. Related Works

The complex computing process of big data affects the real-time performance and accuracy of data processing. Many researchers have proposed different optimization algorithms. Literature [8] proposes a novel deep learning framework for attribute prediction in the wild. This framework not only outperforms the state-of-the-art with a large margin but also reveals valuable facts on learning face representation. Literature [9–11] proposes the reinforcement learning model, which matches behavioral data to explain the observed strategic behavior. The results show that this method is more effective than similar methods. The effect of transfer learning depends to some extent on common factors between learning materials. Literature [12–14] eliminates the training needs of similar learning by learning the transferable structure of scalable image recognition tasks, which significantly reduces the training parameters and the error rate and shortens the training time. In the area of the light field, it is difficult to obtain a complete light field. The huge amounts of data and complex scenes have been obstacles to build light fields.

Many researchers have proposed the concept of light field reconstruction, using the sparsity of the light field to reconstruct the unknown information of the targets from locally known information. Cai et al. [15] proposed a target recognition method based on multiperspective reconstruction, through which the light field data from multiple perspectives are fused and the consistency of the average of all observations is obtained. The experiment shows the robustness and reliability of solving large-scale complex problems with the collaboration of multiple agents. The literature [16–18] uses the features of wavelet transform and multiresolution analysis to effectively suppress the window effect on reconstruction results.

The reconstruction efficiency is improved. In [19], light field imaging is used to solve underwater imaging problems under low-intensity lighting conditions. By using depth convolutional neural networks, the problem of scattering in the light field is effectively solved. By combining light field imaging with structured illumination to perform multiperspective depth measurements, many light field reconstruction methods are proposed [20–23]. The flexible calibration strategy is accordingly designed to determine the mapping coefficients for each light field ray, enabling efficient 3D reconstruction. The angle superresolution method [24–26] is used to capture the light field by using a sparse camera array. It makes use of the compressive sensing reconstruction to collect the dense light field.

Target recognition is an important application in the field of computer vision. Literature [4] proposes a combination method for target tracking with deep learning and preference learning. This method solves the problem of target position and size change during target tracking. It effectively finds out the target object in each frame of the video. Literature [16] introduces light field reconstruction technology to the field of target recognition. The method uses sampled images to reconstruct the light field image and finally tests its validity in the field of target recognition.

To sum up, many researchers have made useful improvements in optimizing algorithms for light field reconstruction. Moreover, they successfully introduced deep learning into light field reconstruction. However, there are still problems: (1) duplicate processing of large amounts of data is constraining real-time performance of target recognition and (2) it is still a challenge to obtain the required data for reconstructing the complete light field.

## 3. Proposed Method

### 3.1. Multiperspective Representation of the Light Field

The Lego Bulldozer of the light field library of the Stanford University is represented as a weight graph *G*=(*v*, *ε*), a given image. The node *v* is the pixels of the image, the light field domain *ε* is the choice of the neighborhood structure, and the weight of the domain is *e*_*ij*_ ∈ *ε*. Define each view field threshold as *C*, then the minimum generalization function *E*(*C*) is

(1) $$\begin{array}{c} E\left(C\right)=\int_{C}-{\left|\nabla I\left(C\left(\varepsilon \right)\right)\right|}^{2}+\alpha {\left|C_{\varepsilon }\left(\varepsilon \right)\right|}^{2}+\beta {\left|C_{\varepsilon \varepsilon }\left(\varepsilon \right)\right|}^{2}\text{d}\varepsilon , \end{array}$$

where ∇*I* is the standard that the threshold value is in the gray gradient area, *α* is a random parameter, and *β* is a weighting function. Specific parameters are added to the threshold, i.e., minimizing functional calculation of the segmentation smooth approximation *u* of the gamma function *I*. Let *I* : *Ω*⟶*ℝ* be the gray value input map in each light field.

(2) $$\begin{array}{c} \underset{\Omega_{t}}{\min}\left\{\frac{1}{2}\overset{k}{\underset{t=0}{\sum }}{\text{per}}_{g}\left(\Omega_{i};\Omega \right)+\overset{k}{\underset{i=0}{\sum }}\int_{\Omega_{i}}f_{i}\left(x\right)\text{d}x\right\}\overset{k}{\underset{i=0}{\cup }}\Omega_{i}. \end{array}$$

In this paper, the convex representation of image segmentation is introduced into multiperspective light field reconstruction [27]. The region *Ω*_*i*_ in the above formula is represented by the labeling function *u* : *Ω*⟶{0,…, *k*}. The *k* binary functions *θ*(*x*)=(*θ*_1_(*x*),…, *θ*_*k*_(*x*)) are equivalent to the multilabel function. Finally, the marker function *u* is recovered from these sequential functions:

(3) $$\begin{array}{c} u\left(x\right)=\overset{k}{\underset{i=1}{\sum }}\theta_{i}\left(x\right). \end{array}$$

**Figure 2:**
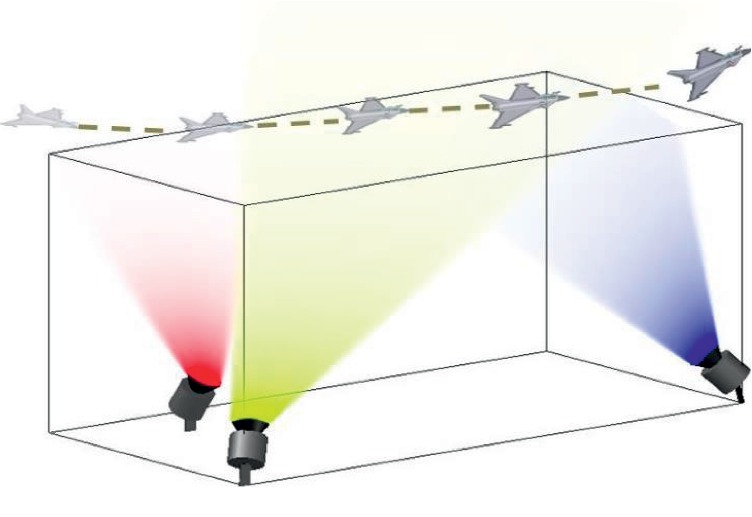
Multiperspective light field 3D representation model. The method uses red, yellow, and blue visible regions to represent the visual range of the multiperspective light field, respectively. In the process of moving the target, the visual range of each perspective and the collaborative relationship between multiple perspectives are embodied.

Therefore, the light field model is divided into many perspectives, as shown in Figure 2.

The multiagent (i.e., multiperspective) method is used to perceive the environment for establishing the source domain, which is also called the instinctive database. It sets the basic action ability for the agent so that the agent is capable of environment interaction and trial-and-error action without training. The multiagent method integrates the collected environmental information and finally completes the establishment of the source domain. According to the content of the environment, the perceptual environment is modeled to train the image. After the image dataset training is completed, the image information is subject to modal analysis. The image is segmented into different categories based on its threshold [28–31]. At present, most segmentation methods are based on pixel-based SoftMax classification. The relationship among pixel points is described by a binary function. Through the relationship, the same labels are assigned to similar pixels and different labels are assigned to pixels with larger gaps so that the image boundary is effectively segmented.

(4) $$\begin{array}{c} \psi_{p}\left(x_{i},x_{j}\right)=u\left(x_{i,}x_{j}\right)\overset{M}{\underset{m=1}{\sum }}W^{m}k_{G}^{m}\left(f_{i},f_{j}\right). \end{array}$$

*u*_*q*_(*x*; *W*) represents the score of the pixel *x* belonging to the category *q*. The probability of the pixel class contained in the image is output by the SoftMax function:

(5) $$\begin{array}{c} S\left(x\right)=p\left(\left.q\right|x,W\right)=\frac{\exp\left(u_{q}\left(x;W\right)\right)}{\sum_{K=1}^{K}\exp\left(u_{q}\left(x;W\right)\right)}, \end{array}$$

where *S*(*x*) represents the result of a light field image segmentation from a single perspective. The light field images captured from multiple perspectives are normalized, and the feature information fusion *u*_*r*_ from multiple perspectives is completed:

(6) $$\begin{array}{c} u_{r}={\left[S{\left(x\right)}_{1},S{\left(x\right)}_{2},\ldots ,S{\left(x\right)}_{N}\right]}^{T}. \end{array}$$

**Figure 3:**
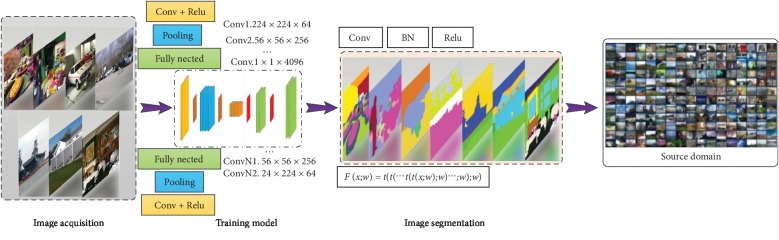
Establishment of the source domain. The acquired image is trained to obtain the state after the image is segmented, and the interpretation of the environment is output.

To add a label to the split body, the label set is called the source domain, as shown in Figure 3. The segmentation method proposed in this paper allows for a wider range of images and lays a good foundation for enhancing the ability of light field reconstruction.

### 3.2. Transfer Reinforcement Learning Algorithm

Duplicate processing of large amounts of data is constraining real-time performance of target recognition. In this paper, the transfer reinforcement learning algorithm is proposed to implement an agent autonomous selection learning model with the ability of analysis, judgment, decision-making, and execution. The target samples captured by the multiple perspectives are traversed in the source domain, and the corresponding learning strategies are selected according to the thresholds of the similar metric models. If the similarity is less than the threshold, reinforcement learning is performed. The decision is adjusted by a small amount of known information and the environment interaction feedback, and finally, the unlabeled sample is iterated with the labeled sample. The new label samples are added to the source domain, and the sample size of the source domain is continuously expanded to improve the environmental cognitive ability and scene-understanding ability. If the similarity is greater than or equal to the threshold, the target-domain sample is directly determined by the feature transfer learning method. This method effectively reduces the number and time of interactions of target-domain samples in the source domain and ensures the real-time performance of target recognition.

#### 3.2.1. Establishment of the Similarity Measurement Model

The perceptual hash algorithm is a mapping of key data in a digital image into a short length sequence. According to the response of machine vision to images in different environments, the perceptual hash algorithm relies on the similarity of the scene to give the corresponding hash value [32]. In this paper, the similarity measures of the perceptual hash algorithm are used to autonomously select reinforcement learning and transfer learning. The specific methods are as follows:

1. The observation image captured by the above multiperspective *u*(*x*) is preprocessed, and the image size is adjusted to 256*∗*256.
2. The feature extraction is performed on the multiperspective image *u*(*x*)={*u*|*x*=1,2,…, *n*}, and the feature vectors *R*={*R*_1_, *R*_2_,…, *R*_*n*_} are obtained, where *R*_*i*_ represents a feature point vector in the graph. The compression of the feature matrix is achieved by summing the multiperspective feature matrices:

(7) $$\begin{array}{c} H\left(i\right)=\overset{n}{\underset{x=1}{\sum }}R_{i,x},\;1\leq \;i\leq \;128. \end{array}$$

In this paper, the feature clustering analysis is used to quantify *H*, and the image information is mapped to 0 or 1 according to the feature clustering threshold *t*. Finally, the hash value *H* is obtained.

(8) $$\begin{array}{c} h\left(i\right)=\left\{\begin{array}{cc} 1, & R_{i,x}\geq t,\\ 0, & R_{i,x}<t. \end{array}\right. \end{array}$$

We use the Hamming distance to judge the similarity among images from multiple perspectives according to the above hash value. Let the multiperspective image be *h*_*n*_, and then the Hamming distance between the image and the feature environment information *h*_0_ is *D*=*h*_*n*_ − *h*_0_. Then, the similarity model of multiperspective images is

(9) $$\begin{array}{c} S=\frac{i-D}{i}. \end{array}$$

Finally, this paper sets the similarity threshold to τ. When the image feature value is less than τ, the model determination features are not similar and the reinforcement learning algorithm is selected; when the image feature value is greater than or equal to τ, the model determination features are similar and the transfer learning algorithm is selected.

#### 3.2.2. Reinforcement Learning Algorithm

Reinforcement learning should try to make a judgment, adjust the previous behavior in the process of interacting with the environment, and then complete the recognition of the target sample of continuous iteration. It is assumed that each step of reinforcement learning has corresponding observations, and the action that can be performed is supported by a small number of label samples in the source domain. The implementation of each step requires a combination of previous actions and observations to take action [33]. Then, the specific steps of reinforcement learning are given below. To simplify the description, the following symbols are defined:

1. *x*_*t*_ ∈ *R*^*m∗n*^ is the corresponding observation image when the target and environment interact at step *t*(*t*=1,2,…, *T*).
2. *a*_*t*_ ∈ ∧ is the action performed by the observation *x*_*t*_, where ∧ is the set of all behaviors under the reinforcement learning rule.
3. *r*_*t*_ is the feedback obtained after performing an action *a*_*t*_ under the observation *x*_*t*_.

(10) $$\begin{array}{c} R_{t}=\overset{T}{\underset{t^{\prime }=t}{\sum }}\gamma^{\left(t^{\prime }=t\right)}\cdot r_{t^{\prime }}, \end{array}$$

where *R*_*t*_ is the collection of all feedback obtained from step *t* to the end time and *γ* is the supervisory budget representing the limitation of computing power and time. In addition, the state *s* at a certain time *t* is *s*_*t*_=(*x*_1_, *a*_1_,…, *x*_*t*−1_, *a*_*t*−1_, *x*_*t*_). Then, the main idea of reinforcement learning is to realize the optimization learning of action state function based on the iteration of *Q*-learning:

(11) $$\begin{array}{c} \left\{\begin{array}{c} Q_{k+1}\left(s_{t},a_{t}\right)=Q_{k}\left(s_{t},a_{t}\right)+\alpha_{k}\cdot \delta_{k},\\ \delta_{k}=r_{t+1}+\gamma \cdot \underset{a^{\prime }\in \wedge }{\max}Q_{k}\left(s_{t+1},a^{\prime }\right)-Q_{k}\left(s_{t},a_{t}\right), \end{array}\right. \end{array}$$

where *α*_*k*_ is the learning rate, *s*_*t*_ and *a*_*t*_ are the states and actions corresponding to step *t*, respectively, and *δ*_*k*_ is the time difference. *a*′ is the action that ∧ can perform in *s*_*t*+1_. The state action value function under the optimal control strategy is obtained as the number of iterations tends to infinity, and the optimal execution strategy in each state is summarized. Finally, the strategy for selecting the optimal executable action in a certain state is to maximize the expected value:

(12) $$\begin{array}{c} Q^{*}\left(s,a\right)={Ε}_{s^{\prime }∼\xi }\left(r+\gamma \cdot \underset{a^{\prime }}{\max}Q^{*}\left(s^{\prime },a^{\prime }\right)\left|s,a\right.\right), \end{array}$$

where *ξ* is the environment, *s*′ is the state after *s* performs an action *a*, and *a*′ is all possible actions of the state *s*′. The above formula uses the expectation to analyze the state action value function and uses *Q*(*s*, *a*, *θ*) ≈ *Q*^*∗*^(*s*, *a*) to estimate the state action function. In reinforcement learning, the *Q*-network implements parameter updation by minimizing the objective function.

(13) $$\begin{array}{c} L_{k}\left(\theta_{k}\right)={Ε}_{s,a∼\rho \left(\cdot \right)}{\left[y_{k}-Q\left(s,a,\theta_{k}\right)\right]}^{2}. \end{array}$$

Here, *ρ*(*s*, *a*) is the probability distribution of the state *s* and behavior *a*, and *y*_*k*_ is the target output corresponding to the *k*th iteration and is given by

(14) $$\begin{array}{c} y_{k}={Ε}_{s^{\prime }∼\xi }\left(r+\gamma \cdot \underset{a\prime }{\max}Q\left(s\prime ,a\prime ,\theta_{k-1}\right)\left|s,a\right.\right). \end{array}$$

Let all subjects evaluate the results. If the objective function obtained by the subject under various constraints is smaller, the subject should receive a larger reward:

(15) $$\begin{array}{c} R^{\prime }\left(s_{k}^{\prime },s_{k+1}^{\prime },a_{k}^{\prime }\right)=\left\{\begin{array}{cc} \frac{W}{f_{\text{Best}}}, & \left(s_{k}^{\prime },a_{k}^{\prime }\right)\in y_{k},\\ 0, & \text{otherwise}, \end{array}\right. \end{array}$$

where *f*_Best_ represents the fitness function of the optimal state at the *k*th iteration and *W* is a positive constant; the smaller the value of the objective function, the larger the reward value. In this paper, based on the experience of previous learning, the prompt for the next target environment interaction is *e*_*t*_. Assume that the number of steps at the end of reinforcement learning is *N* and the set of reviews for experience is *D*=[*e*_1_, *e*_2_,…, *e*_*N*_]. We feed *D* back to the source domain, increasing the amount of data of known samples in the source domain. Therefore, the behavior state action value in the transfer reinforcement learning algorithm is changed to TRL(*s*, *a*, *θ*)⟶TRL(*φ*(*s*), *a*, *θ*). *X* is the feature learning in the transfer reinforcement learning algorithm, so the state of the step *t* in the transfer reinforcement learning is *s*_*t*+1_=(*s*_*t*_, *a*_*t*_, *x*_*t*+1_). Finally, the modified equation for the interaction of the target environment in reinforcement learning is obtained:

(16) $$\begin{array}{c} \left\{\begin{array}{c} D⟶\bar{D}=\left[{\bar{e}}_{1},{\bar{e}}_{2},\ldots ,{\bar{e}}_{N}\right],\\ {\bar{e}}_{t}=\left(\varphi \left(s_{t}\right),a_{t},r_{t},\varphi \left(s_{t+1}\right)\right) \end{array}\right.. \end{array}$$

**Figure 4:**
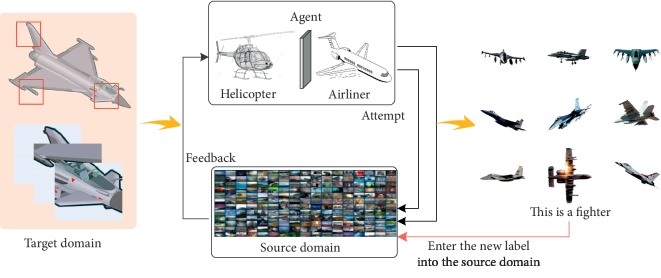
Reinforcement learning of unlabeled samples in the target domain.

The red box represents the target information captured from multiple perspectives. The local information captured from each perspective is preliminarily judged as a helicopter or an airliner, and the target information and the environment keep interacting to conclude that the target is a fighter. We will input new label samples into the source domain after reinforcement learning, continuously update the source-domain sample data, expand the label sample size, and improve the environmental cognitive ability and scene-understanding ability, as shown in Figure 4.

#### 3.2.3. Transfer Learning Algorithm

The multiperspective image is taken as the target domain. By comparing with the label samples in the source domain, the feature transfer method in the transfer learning is selected to recognize the environment of the multiperspective samples [34, 35]. Let the source domain after reinforcement learning be *D*_*s*_={*x*_*s*_*i*__, *y*_*s*_*i*__}. The information captured by multiple perspectives is the target domain *D*_*T*_={*x*_*T*_*i*__}. As *Y*_*s*_ continues to increase, there will be more and more *Y*_*T*_ in *D*_*T*_ similar to *Y*_*s*_. In order to avoid repeated calculation of similar feature data, this paper adopts PCA based on the transfer learning algorithm. It is known that *φ* is feature learning; then, *φ*(*X*_*s*_) and *φ*(*X*_*T*_) represent *X*_*s*_ and *X*_*T*_ data features, respectively. The unmarked data *x*_*T*_*i*__ in *D*_*T*_ are mapped to the feature space to obtain a new representation *φ*(*X*_*T*_*i*__). In transfer learning, the optimal learning state is the minimum expected risk. Therefore, the optimal model for learning the target domain is proposed:

(17) $$\begin{array}{c} \theta =\arg\min\underset{i=1}{\sum }P\left(D_{T}\right)l\left(x,y,\theta \right), \end{array}$$

where *P*(*D*_*T*_) is the probability of edge distribution in the target domain and *l*(*x*_*i*_, *y*_*i*_, *θ*) is the loss function. When *P*(*D*_*S*_) ≠ *P*(*D*_*T*_), the above model is optimized to enhance the generalization ability of the transfer learning target domain:

(18) $$\begin{array}{c} \theta =\arg\min\underset{i=1}{\sum }\frac{P\left(D_{T}\right)}{P\left(D_{S}\right)}P\left(D_{S}\right)l\left(x,y,\theta \right),\\ \approx \arg\min\overset{ns}{\underset{i=1}{\sum }}\frac{P_{T}\left(x_{T_{i}},y_{T_{i}}\right)}{P_{S}\left(x_{S_{i}},y_{S_{i}}\right)}l\left(x_{S_{i}},y_{S_{i}},\theta \right). \end{array}$$

According to the probability distribution function of the source domain and the target domain, the maximum embedded space between them can be obtained:

(19) $$\begin{array}{c} \text{Dist}\left(\varphi \left(X_{S}\right),\varphi \left(X_{T}\right)\right)=\left\|\frac{1}{n_{S}^{2}}\overset{n_{S}}{\underset{i,j=1}{\sum }}k\left(x_{S_{i}},x_{S_{j}}\right)+\frac{1}{n_{T}^{2}}\overset{n_{T}}{\underset{i,j=1}{\sum }}k\left(x_{T_{i}},x_{T_{j}}\right)-\frac{2}{n_{S}n_{T}}\overset{n_{S},n_{T}}{\underset{i,j=1}{\sum }}k\left(x_{S_{i}},x_{T_{j}}\right)\right\|. \end{array}$$

In order to obtain the optimal eigenvalues in the maximum embedded space, we use the kernel matrices *K*_*s*_ and *K*_*T*_ to represent the feature matrix $K=\left[\begin{array}{cc} K_{S,S} & K_{S,T}\\ K_{T,S} & K_{T,T} \end{array}\right]$ on the source- and target-domain data. In this section, $\overset{˜}{W}$ of (*n*_*S*_+*n*_*T*_) × *m* matrix transformation is used to map the kernel matrix to the *m*-dimensional space to obtain the resultant kernel matrix as follows:

(20) $$\begin{array}{c} \tilde{K}=\left(KK^{-1/2}\tilde{W}\right)\left({\tilde{W}}^{T}K^{-1/2}K\right)=KWW^{T}K. \end{array}$$

Then, the corresponding kernel matrix value between *x*_*i*_ and *x*_*j*_ is

(21) $$\begin{array}{c} \overset{˜}{K}\left(x_{i},x_{j}\right)=K_{x_{i}}^{T}WW^{T}K_{x_{j}}. \end{array}$$

The transfer learning mapping data *x*_*S*_*i*__ and *x*_*T*_*j*__ are obtained by calculating *X*_*S*_′=[*K*_*S*,*S*  _  *K*_*S*,*T*_]*W* and *X*_*T*_′=[*K*_*T*,*S*_*K*_*T*,*T*_]*W*.

**Figure 5:**
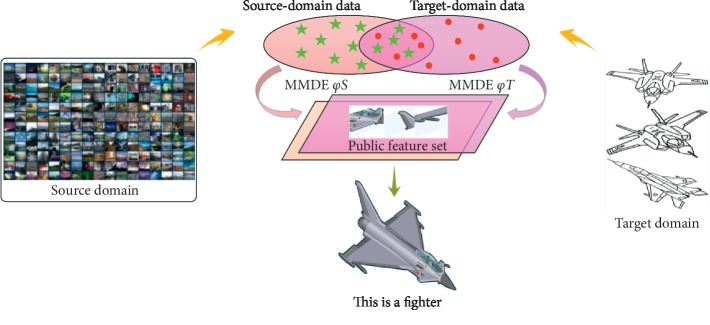
Feature transfer learning with tag samples in the target domain. The source domain and the target domain are mapped to the label samples by dimension reduction, and the PCA is used to project the same type labels into the common feature set. The sample tag of the target domain is output by the feature migration learning method.

The transfer learning for target features is to map the labeled image samples captured from each perspective in the target domain and the labeled samples in the source domain to the common feature subspace at the same time. Through the feature comparison between the tag samples in the source domain and those in the target domain, the target is recognized as a fighter. This method reduces the computation and recognition time of sample recognition in the target domain, and the specific process is shown in Figure 5. To sum up, the specific method of transfer reinforcement learning is shown in Algorithm 1.

#### 3.2.4. Multiperspective Light Field Reconstruction Method Based on Transfer Reinforcement Learning

**Figure 6:**
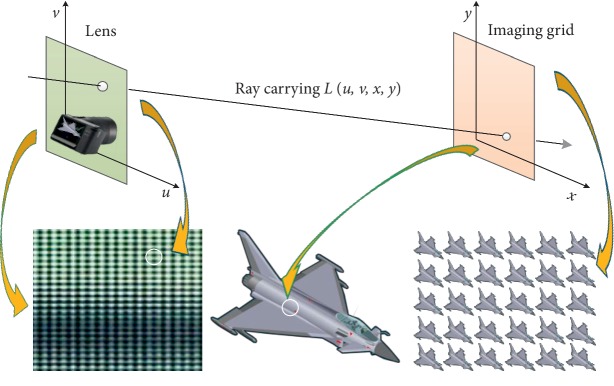
Light field imaging model. Taking the aircraft model as an example, the green plane is the camera microlens imaging surface and the orange plane is the imaging grid surface.

According to the establishment of the multiperspective representation model, the multiperspective light field is reconstructed using the transfer reinforcement learning algorithm. The original light field image is a (*x*, *y*) ray imaging grid. Each image represents that the light reaches a microlens on the imaging surface, which is from different (*u*, *v*) positions of the main lens, and it is shown in Figure 6.

The original image is composed of a series of pixels, each of which is microlens imaged. Because the aperture is limited, each microlens has a certain field of view, and there is a certain parallax among the different microlenses.

(22) $$\begin{array}{c} E_{F}\left(x,y\right)=\frac{1}{F^{2}}\iint L_{F}\left(x,y,u,v\right)\cos^{4}\;\theta \text{ d}u\text{ d}v, \end{array}$$

where *L*_*F*_(*x*, *y*, *u*, *v*) is the light field parameter from the target plane *F* and cos  *θ* is the attenuation factor due to the optical halo effect. The multiperspective feature information fusion *u*(*r*) and the feature learning *φ* in transfer reinforcement learning are introduced into the light field reconstruction process. Then, the point imaging function can be obtained in any plane letting (*x*, *y*, *u*, *v*)⟶*φ*(*x*′, *y*′, *u*(*r*), *v*′).

(23) $$\begin{array}{c} E_{\left(\alpha ,F\right)}\left(x^{\prime },y^{\prime }\right)=\frac{1}{\alpha^{2}F^{2}}\iint L_{F}\left(u\left(1-\frac{1}{\alpha }\right)+\frac{x^{\prime }}{\alpha },v\left(1-\frac{1}{\alpha }\right)+\frac{y^{\prime }}{\alpha },u,v\right)\text{d}u\text{d}v. \end{array}$$

**Figure 7:**
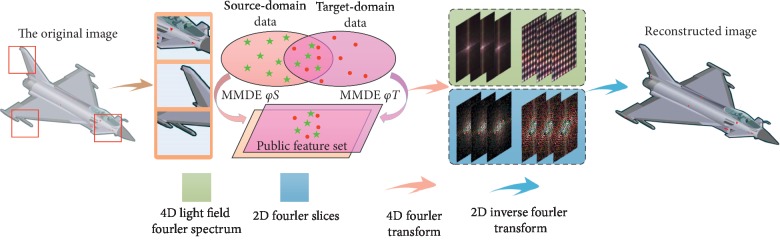
Multiperspective light field reconstruction based on transfer reinforcement learning.

According to the similarity threshold of different scenes, the algorithm in this paper autonomously chooses different models of transfer reinforcement learning (i.e., transfer learning or reinforcement learning) to reconstruct the multiperspective light field. Based on the algorithm, the frequency-domain information of images can be obtained by 4D Fourier transform of the multiperspective light field. And then the center slice and the inverse wavelet transform are used to obtain the reconstructed light field image of each perspective, as shown in Figure 7.

## 4. Experimental Results and Analyses

**Figure 8:**
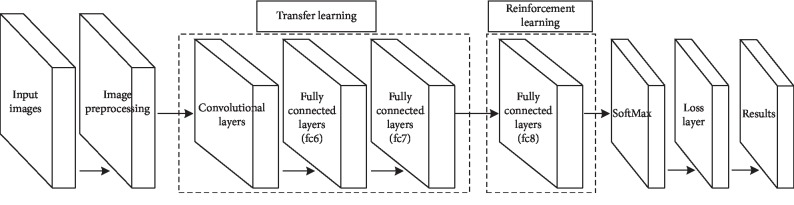
Multiperspective light field reconstruction method training model network.

PASCAL VOC provides a standard set of excellent datasets for image recognition and classification. Among them, the VOC2007 dataset contains 9963 labeled images, consisting of trains, fuel consumption, and tests, with a total of 24640 labeled objects. The VOC2012 dataset is an upgraded version of the VOC2007 dataset, with a total of 11,530 images. The VOC2012 dataset is divided into 20 categories, such as people, birds, dogs, airplanes, and cars. In order to ensure the objectivity and persuasiveness of the experiment, the training set samples used for animal detection, vehicle detection, and human detection are 6800 images with a pixel size of 500 *∗* 332. The test set samples are 1200 images with a pixel size of 500 *∗* 375. In this paper, the detection rate is selected as a performance measure for statistical significance test analysis. The detection rate refers to the recognition ratio between the target and the background in the recognition window. We use TensorFlow on the Windows 10 system for experimental simulation. The simulation calculation runs on a small server with an E5-2630 v4 CPU, the main frequency of 2.2 GHz, and a memory of 32 GB. The multiperspective light field reconstruction method training model network structure is shown in Figure 8.

This section selects several representative data to describe and analyze the target recognition. The experimental data of target recognition are divided into three categories: animals, vehicles, and humans. The test data are sorted according to the number of targets and the complexity of scenarios. The algorithm in this paper identifies targets simultaneously with existing RPN [36], Fast R-CNN [37], YOLO 9000 [38], SSD [39], and R-CNN [40] algorithms.

**Figure 9:**
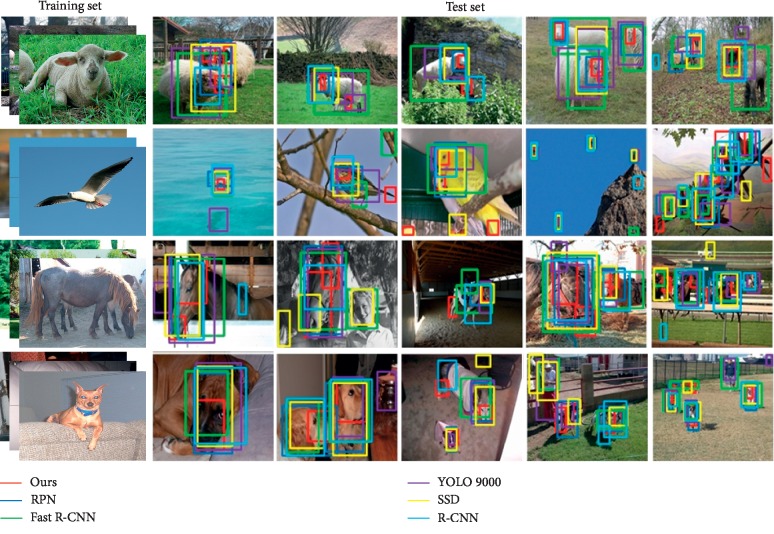
Recognition results of animal samples.

Figure 9 shows the animal samples for the test dataset, which include sheep, bird, horse, and dog. In the sheep recognition results, we can see that the algorithm has good results in the second, third, and fifth tests. It includes target occlusion and small targets at a distance. In the bird recognition results, we can see from the fifth test data that the algorithm in this paper has accurately identified each target in the graph. This shows that the algorithm is suitable for multitarget recognition. In the horse recognition results, we can conclude from the third test data that the algorithm can effectively avoid the impact of the light problem on target recognition. In the dog recognition results, we can see the effectiveness of the algorithm in multitarget recognition from the results of the fourth and fifth test data. From Table 1, it can be seen that the average recognition accuracy of the algorithm in the detection of the animal dataset is 78%, which is higher than that of other algorithms.

**Figure 10:**
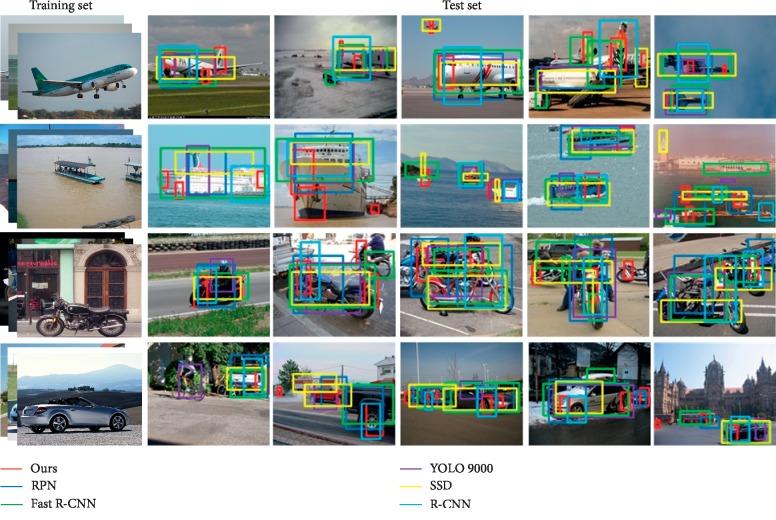
Recognition results of vehicle samples.

Figure 10 shows samples of vehicles for the test set, which include daily vehicles such as airplanes, ships, and cars. In the fourth test data of airplanes, it is reflected that all algorithms are less robust to target local information recognition under cluttered background. However, the algorithm identifies the greatest number of targets compared to other algorithms. In the second test data of the airplanes, the algorithm effectively solves the problem of insufficient light. However, in the identification result of the ship, the fifth test data are large due to the fog at sea, and all algorithms are not recognized for distant targets. It can be seen from the recognition results of motorcycles and cars that when the targets are seriously overlapped, other algorithms mentioned in this paper cannot accurately identify them. From Table 2, it can be seen that the average recognition accuracy of the algorithm in the detection of the vehicle dataset is 76.9%, which is higher than that of other algorithms.

This paper assumes *H*_0_ : *μ*_Others_=*μ*_Ours_, which proves that there is no difference between the two methods for the same target recognition. *H*_*A*_ : *μ*_Others_ ≠ *μ*_Ours_, which proves that there is a difference between the two methods when identifying the same target. In these relations, Others represent the existing algorithms and Ours represents the algorithm in this paper. In order to verify the effectiveness of the algorithm in this paper, we set the significance level to the international universal level *a*=0.05. That is, the confidence interval is 95%. The sample is *n*, and d*f*=2(*n* − 1). Therefore, the standard deviation of the mean is $S_{\bar{\text{Others}}-\bar{\text{Ours}}}=\sqrt{\left({S_{\text{Others}}}^{2}/n\right)+\left({S_{\text{Ours}}}^{2}/n\right)}$, and the statistic $t=\left(\left|\bar{\text{Others}}\right|-\bar{\text{Ours}}\right)/\left(S_{\bar{\text{Others}}-\bar{\text{Ours}}}\right)$.

**Figure 11:**
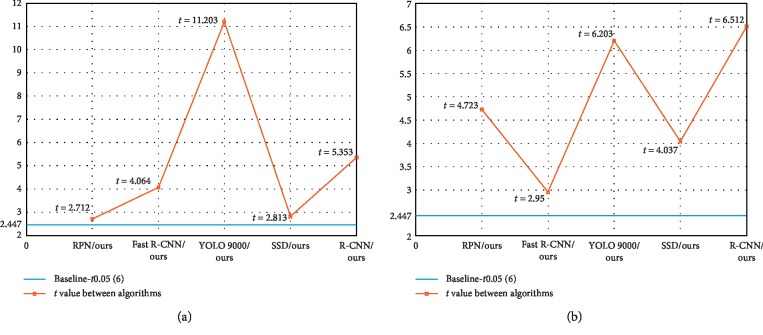
Analysis chart of the statistical significance test of each target algorithm in the animal dataset (a) and vehicle dataset (b).

We set the confidence interval to 95% and the sample category to *n*=4. Therefore, the benchmark for the statistical significance test is 2.447. As can be seen from Figure 11(a) and 11(b), the statistical significance test indicators of other algorithms and the algorithm in this paper are higher than 2.447. Then, we can judge the hypothesis. There are statistically significant differences between our algorithm and other algorithms in animal and vehicle datasets.

**Figure 12:**
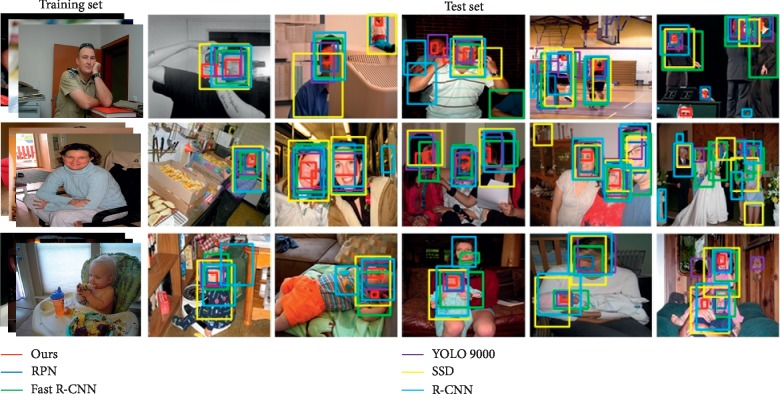
Recognition results of human samples.

Figure 12 shows a test dataset of human samples that include men, women, and baby. In the male identification results, both the second and fifth test data showed interference items that were not physical. Therefore, our algorithm, RPN algorithm, and SSD algorithm all identify errors in the second test data. However, in the fourth test data, the motion occlusion problem was effectively solved and all targets were correctly identified. In the female recognition results, our algorithm successfully distinguishes the males in the fourth test data. Because the fifth test data have many tasks, the features of individual characters are not obvious. Therefore, the algorithms mentioned in this paper have different errors in the target recognition process. In the baby's recognition results, all algorithms are accurately identified in both the first and second test data. In the third and fourth test data, the SSD algorithm and the R-CNN algorithm identify objects outside the target. However, in the fifth test data, all algorithms cannot be accurately identified because the scene is too complicated and the target is small. As can be seen from Table 3, when detecting the baby dataset, the recognition rate of the RPN algorithm and the algorithm in this paper reached 71.4% at the same time. In the male dataset and female dataset, the recognition accuracy of the algorithm in this paper is 72.5%, which is higher than that of other algorithms.

**Figure 13:**
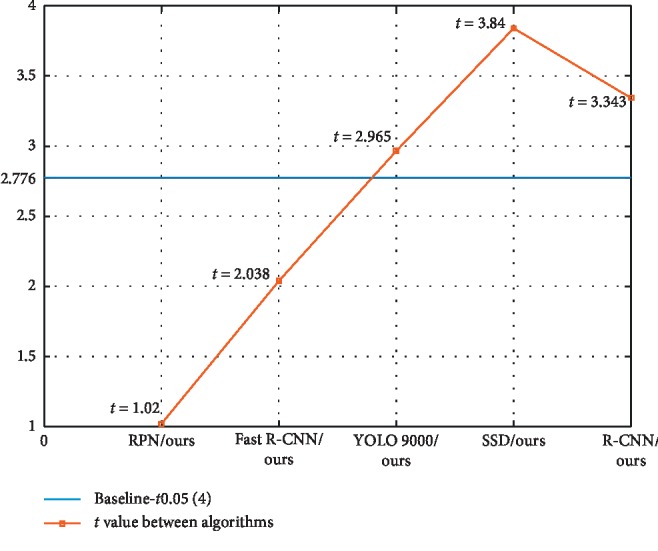
Analysis chart of the statistical significance test of each target algorithm in the human dataset.

Since the sample category of the human dataset is *n*=3, the benchmark for the statistical significance test is 2.776. As can be seen from Figure 13 and Table 3, the accuracy of the proposed target recognition algorithm on the human dataset is 72.5%, which is higher than that of RPN and Fast R-CNN. However, the statistical significance test indicators are lower than the baseline. This shows that the hypothesis test is valid, and the recognition results of the algorithm in this paper are not significantly different from those of the PRN and Fast R-CNN algorithms. Compared with those of YOLO 9000, SSD, and R-CNN algorithms, the statistical significance test indicators of the algorithm in this paper are higher. This shows that there are obvious differences between the algorithm in this paper and them.

Based on the experimental results of target recognition and statistical significance test analysis, the algorithm in this paper fully reflects the advantages of multiple perspectives in target recognition. In target recognition, the larger the boundary box, the less accurate the target recognition result. When detecting any feature of the target, the algorithm uses several small red boxes to label it. Therefore, the above target recognition results verify the effectiveness of the proposed algorithm.

## 5. Conclusions

Duplicate processing of target data and lack of required data affect the accuracy and real-time performance of reconstruction of the complete light field. In view of the problems, this paper proposes the transfer reinforcement learning method for multiperspective light field reconstruction. According to the similarity threshold, reinforcement learning or transfer learning can be selected independently. Our algorithm effectively solves the repeated calculation of the same data and shortens the time of multiperspective light field reconstruction. The experimental result shows that the transfer reinforcement learning algorithm is better than other algorithms in target recognition. In the experiment, the recognition efficiency of the algorithm in this paper makes it not ideal for recognition in the foggy environment. In the future work, we will introduce the GAN to transfer learning. We will use the characteristics of the GAN to generate data and enhance data to process foggy images and increase their target recognition accuracy.

## Figures and Tables

**Algorithm 1:**
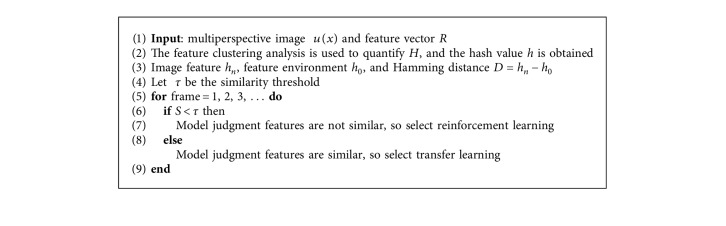
Transfer reinforcement learning.

**Table 1:** Detection rate evaluation report form for different algorithms in the animal dataset.

	Sheep, average	Bird, average	Horse, average	Dog, average	Animal dataset, average	Standard deviation
RPN	71.7	75.2	74.5	77.4	74.7	2.35
Fast R-CNN	71.6	73.6	73	76.4	73.7	2.02
YOLO 9000	71.7	72.4	73.3	73.1	72.6	0.73
SSD	69.3	71.3	71.6	73.6	71.5	1.76
R-CNN	71.6	71.7	72.3	76.4	73	2.29
**Ours**	**77.2**	**78.2**	**78.7**	**77.8**	**78**	0.63

VOC2012 animal dataset detected accuracy (%). Average: average recognition rate of five samples in each dataset.

**Table 2:** Detection rate evaluation report form for different algorithms in the vehicle dataset.

Method	Airplane, average	Boat, average	Motorcycle, average	Car, average	Vehicle dataset, average	Standard deviation
RPN	72.4	72.5	74.3	72.3	72.9	0.95
Fast R-CNN	74.4	74.7	74.8	71.7	73.9	1.48
YOLO 9000	72.8	71.3	70.4	70	71.1	1.24
SSD	74.9	71.1	72.3	71.7	72.5	1.67
R-CNN	70.9	72.7	70.4	71	71.3	1
**Ours**	**78.1**	**76.8**	**77.8**	**75**	**76.9**	1.4

VOC2012 vehicle dataset detected accuracy (%). Average: average recognition rate of five samples in each dataset.

**Table 3:** Detection rate evaluation report form for different algorithms in the human dataset.

Method	Male, average	Female, average	Baby, average	Human dataset, average	Standard deviation
RPN	71.8	72.3	**71.4**	71.8	0.45
Fast R-CNN	71.3	71.3	71.1	71.2	0.12
YOLO 9000	70	71.1	69.9	70.3	0.67
SSD	70	69.8	70.3	70	0.25
R-CNN	69.8	70.7	70.1	70.2	0.46
**Ours**	**73.6**	**72.5**	**71.4**	**72.5**	1.1

VOC2012 human dataset detected accuracy (%). Average: average recognition rate of five samples in each dataset.

## Data Availability

The dataset contains confidential information such as the performance parameters of the aircraft model and cannot be released.
